# Identification and Characterization of OXA-232-Producing Sequence Type 231 Multidrug Resistant Klebsiella pneumoniae Strains Causing Bloodstream Infections in China

**DOI:** 10.1128/spectrum.02607-22

**Published:** 2023-03-22

**Authors:** Tao Chen, Hongyun Xu, Yunbo Chen, Jinru Ji, Chaoqun Ying, Zhiying Liu, Hao Xu, Kai Zhou, Yonghong Xiao, Ping Shen

**Affiliations:** a State Key Laboratory for Diagnosis and Treatment of Infectious Diseases, National Clinical Research Center for Infectious Diseases, Collaborative Innovation Center for Diagnosis and Treatment of Infectious Diseases, the First Affiliated Hospital, Zhejiang University School of Medicine, Hangzhou, Zhejiang, China; b Department of Clinical Laboratory, the Second People's Hospital of Yunnan province, Kunming, Yunnan, China; c First Affiliated Hospital of Southern University of Science and Technology (Shenzhen People’s Hospital), Shenzhen, Guangdong, China; d Jinan Microecological Biomedicine Shandong Laboratory, Jinan, Shandong, China; Emory University School of Medicine

**Keywords:** OXA-232, multidrug resistant, ST231, *Klebsiella pneumoniae*, dissemination

## Abstract

Klebsiella pneumoniae, a notorious pathogen for opportunistic health care-associated infections, represents increasing multidrug resistance, particularly to carbapenems. OXA-232 carbapenemase, as a variant of OXA-48, has been increasingly reported worldwide. ST231, an epidemic, multidrug resistant (MDR) K. pneumoniae clone in south and southeast Asia, has been found in other regions, including Europe. In the study, five OXA-232 carbapenemase-producing Klebsiella pneumoniae isolates, four of which belong to sequence type 231 (ST231) and one of which belongs to ST15, were isolated from two hospitals in China. All isolates displayed a MDR phenotype, being susceptible to only polymyxin B and colistin, and the *bla*_OXA-232_ gene was located on a ColKP3-type nonconjugative plasmid of 6.1 kb. A phylogenetic analysis of the global ST231 K. pneumoniae isolates (*n* = 231) suggested that the four ST231 isolates from this study gathered with strains from south Asia (especially India), indicating that the emerging Chinese ST231 clone was more closely related to south Asia isolates and might have spread from south Asia, where ST231 was a successful epidemic clone. Virulence assays suggested that the four ST231 strains were not highly virulent, as they displayed significantly lower virulence potential, compared with a ST23 K1 hypervirulent isolate in a G. mellonella infection and in mouse intraperitoneal infection models, although three ST231 strains harbored a plasmid-borne aerobactin-encoding *iuc* gene cluster. This is the first report of ST231 K. pneumoniae clinical strains bearing *bla*_OXA-232_ in China, and it highlights the emergence of the ST231 clone causing bloodstream infections in a health care setting as well as calls attention to the transmission of this emerging clone in China.

**IMPORTANCE** OXA-232 carbapenemase, being a vital resistance mechanism against carbapenems, has recently been increasingly reported. In China, the identified OXA-232-producing K. pneumoniae isolates almost belonged to ST15 and were not hypervirulent, despite harboring a virulence plasmid. Here, we report the first occurrence in China of a MDR OXA-232-producing K. pneumoniae ST231 clone that is an epidemic ST type in south and southeast Asia. A phylogenetic analysis indicated that this emerging Chinese ST231 clone was more closely related to Indian isolates. The occurrence of this clone may have been driven through the transnational importation of Indian ST231 K. pneumoniae clones. Moreover, this study is the first to assess the virulence potential of ST231 clones that have never been estimated in previous studies. While the high burden of MDR K. pneumoniae is concerning, genomic surveillance can shed light on the transmission chains of novel MDR clones, and active surveillance should be enforced to restrict the spread of MDR isolates.

## INTRODUCTION

The carbapenem-hydrolyzing class D carbapenemases are primary causes of carbapenem resistance among the Enterobacterales and A. baumannii isolates (1). OXA-48, a class D carbapenemase, was first identified in a clinical Klebsiella pneumoniae isolate in 2004 (2). Since then, OXA-48-producing Enterobacteriaceae have been increasingly reported worldwide (3). To date, several variants of OXA-48, differing by only a few amino acids and displaying similar enzymatic profiles, have been identified. The common acquired OXA-48-type carbapenemases include OXA-48, OXA-181 (4), and OXA-232 (5). OXA-232, which was first reported in Escherichia coli and K. pneumoniae strains that were isolated from three French patients traveling to India in 2013, differs from OXA-48 and OXA-181 by five amino acid substitutions and one amino acid substitution, respectively. Similar to OXA-48, OXA-232 has reduced hydrolytic activity against carbapenems but exhibits higher hydrolytic activity against penicillins.

To date, OXA-232-producing K. pneumoniae isolates have been identified worldwide, and the diversity of sequence types (STs) of these isolates has been found, including ST14, ST15, ST16, ST17, ST76, ST147, ST231, ST307, ST395, and ST437 (6–10). In China, the identified OXA-232-producing K. pneumoniae isolates primarily belonged to ST15 and carried a 6.1-kp ColKP3 plasmid bearing the *bla*_OXA-232_ gene (6, 11, 12). Previously, multidrug resistant (MDR) K. pneumoniae ST231 isolates that were coproducing OXA-232, namely, the extended-spectrum-beta-lactamase CTX-M-15 and the 16S rRNA methyltransferase RmtF, had emerged as successful epidemic clones in south and southeast Asia, with related isolates being reported in India, Singapore, and Brunei Darussalam (13–15). Here, we report on the incidence of the clonal dissemination of the OXA-232-producing MDR K. pneumoniae of ST231, which represents the first detection of ST231 K. pneumoniae clinical strains bearing *bla*_OXA-232_ in China. Moreover, we investigated the genomic features and clonal origins of these ST231 K. pneumoniae isolates and determined for the first time the virulence potential of ST231 strains.

## RESULTS

4 patients, aged 50 to 68 years, were admitted to the same intensive care unit at the Second People's Hospital of Yunnan Province between December of 2018 and November of 2019. Additionally, a patient, aged 59 years, was admitted to the hematology department at the First Affiliated Hospital of Zhejiang University, School of Medicine, in October of 2019. All of the patients suffered from bloodstream infections, and three were diagnosed with pneumonia. All of the patients were treated with carbapenems or beta-lactam/beta-lactamase inhibitor combinations, and three of them died (Table 1). Moreover, none of the patients reported recent travel abroad (from 2018 to 2019). Five nonduplicate, OXA-232-producing K. pneumoniae strains were isolated from the blood samples of the five patients. The results of antimicrobial susceptibility testing indicated that all of the clinical isolates were susceptible to polymyxin B and colistin but were resistant to other antimicrobial agents (except KP100073, which was susceptible to chloramphenicol), thereby displaying a multidrug resistance phenotype (Table 2).

**TABLE 1:** Clinical features of patients infected with OXA-232 carbapenemase-producing K. pneumoniae isolates^*a*^

Patient no.	Collected isolate	Date of isolation	Specimen	Province of isolation	Age	Sex	Underlying condition	Days of hospital stay before infection/total days	Invasive procedure	Antimicrobial therapy	Status at hospital discharge
1	KP88385	2018.12.12	Blood	Yunnan	50	Male	Acute diffuse peritonitis, pneumonia	4/28	None	CSL	Discharged for further treatment
2	KP100073	2019.06.26	Blood	Yunnan	55	Male	Head injury	24/48	endotracheal intubation	LVX, MEM, TGC	Died
3	KP108505	2019.09.24	Blood	Yunnan	60	Female	Diabetes, cholangiocarcinoma, pneumonia	36/44	PICC, endotracheal intubation	CSL, IPM, TGC	Died
4	KP114692	2019.11.23	Blood	Yunnan	68	Male	Heart failure, renal failure, pneumonia	71/72	PICC, endotracheal intubation	MFX, IPM, MEM, LVX, CSL	Died
	KP106903	2019.10.11	Blood	Zhejiang	59	Female	AML, myelosuppression	3/22	None	TZP, IPM, TGC, CSL, MFX	Recovered

a PICC, peripherally inserted central catheter; AML, acute myelogenous leukemia; CSL, cefoperazone-sulbactam; LVX, levofloxacin; MEM, meropenem; TGC, tigecycline; IPM, imipenem; MFX, moxifloxacin; TZP, piperacillin-tazobactam.

**TABLE 2:** Susceptibility of *bla*_OXA-232_-producing K. pneumoniae to commonly used antibiotics^*a*^

Isolates	ST type	CAZ	CXM	FEP	IPM	MEM	CIP	AMP	AMK	GEN	CSL	SXT	CHL	POLB	CST	TGC
KP88385	ST231	>64	>64	>64	8	32	1	>128	>128	>128	>128	>32/608	>128	1	0.5	8
KP100073	ST231	>64	>64	>64	4	32	1	>128	>128	>128	>128	>32/608	16	1	1	4
KP108505	ST231	>64	>64	>64	4	32	1	>128	>128	>128	>128	>32/608	>128	2	2	16
KP114692	ST231	>64	>64	>64	8	32	1	>128	>128	>128	>128	>32/608	>128	1	1	8
KP106903	ST15	>64	>64	>64	4	16	1	>128	>128	>128	>128	>32/608	>128	2	2	8

a CAZ, ceftazidime; CXM, cefuroxime; FEP, cefepime; IPM, imipenem; MEM, meropenem; CIP, ciprofloxacin; AMP, ampicillin; AMK, amikacin; GEN, gentamicin; CSL, cefoperazone-sulbactam; SXT, sulfamethoxazole-trimethoprim; CHL, chloramphenicol; POLB, polymyxin B; CST, colistin; TGC, tigecycline. Resistance to antibiotics is indicated by shading.

At first, the draft genomes of five K. pneumoniae strains were constructed using short-read data. The draft genomes revealed that four isolates (KP88385, KP100073, KP108505, and KP114692) belonged to ST231 and serotype KL51, whereas one isolate (KP106903) belonged to ST15 and KL112. Because ST231 K. pneumoniae was undiscovered in China, the four ST231 isolates were subject to MinION sequencing to obtain the long-read data for genomic characterization. We performed hybrid assembly for these strains using Illumina and Nanopore reads. The assembly results showed 4 to 7 contigs, an estimated genome length of 5,637,960 to 5,746,525 bp, and an average G+C content of 56.94% to 57.02% among these four ST231 isolates. The N_50_ lengths of the four assemblies, which were defined as the shortest sequence lengths at 50% of the genome, were between 5,303,591 and 5,489,177 bp. A pairwise SNP analysis of the four ST231 strains, based on their complete genomes, showed that their core genomes differed by only a few SNPs (*n* = 9 to 16), suggesting that the ST231 strains were closely related. All four of the ST231 isolates were found to harbor genes encoding β-lactamases (CTX-M-15, SHV-212, and CTX-M-186) and a carbapenemase (OXA-232), with three of them (except KP88385) also possessed the β-lactamase gene *bla*_TEM-181_. The *iuc5* gene cluster, encoding the siderophore aerobactin, which was associated with invasive disease and was common among hypervirulent K. pneumoniae clones that lead to severe community-associated infections, such as liver abscess and pneumonia, was detected in three of the ST231 isolates (except KP100073), and the aerobactin allele *iuc1* was only detected in KP106903. Furthermore, the regulator gene of the mucoid phenotype *rmpA2* was solely found in KP106903 (Table 3). Nevertheless, similar to the four ST231 isolates, KP106903 did not display a hypermucoviscous phenotype that was detectable as a positive “string test” result.

**TABLE 3:** The characteristics of 5 *bla*_OXA-232_-producing K. pneumoniae genomes

Isolates	ST type	Approximate size of genome	Plasmid replicons	β-lactamases and carbapenemases	Aerobactin synthesis locus (*iuc*) allele	*rmpA/A2*
KP88385	ST231	5698589 bp	ColKP3, IncFIA, IncFII, IncFIB, IncFII(K)	CTX-M-15, SHV-212, CTX-M-186, OXA-232	*iuc5*	None
KP100073	ST231	5637960 bp	ColKP3, IncFIB, IncFII(K)	CTX-M-15, SHV-212, TEM-181, CTX-M-186, OXA-232	None	None
KP108505	ST231	5710289 bp	ColKP3, IncFIA, IncFII, IncFIB, IncFII(K)	CTX-M-15, SHV-212, TEM-181, CTX-M-186, OXA-232	*iuc5*	None
KP114692	ST231	5746525 bp	ColKP3, IncFIA, IncFII, IncFIB, IncFII(K)	CTX-M-15, SHV-212, TEM-181, CTX-M-186, OXA-232	*iuc5*	None
KP106903	ST15	5751685 bp	ColKP3, IncHI1B, IncFIB, IncFII(K), ColRNAI, repB, Col440I	CTX-M-15, SHV-106, TEM-181, OXA-232	*iuc1*	*rmpA2*

**FIG 1:**
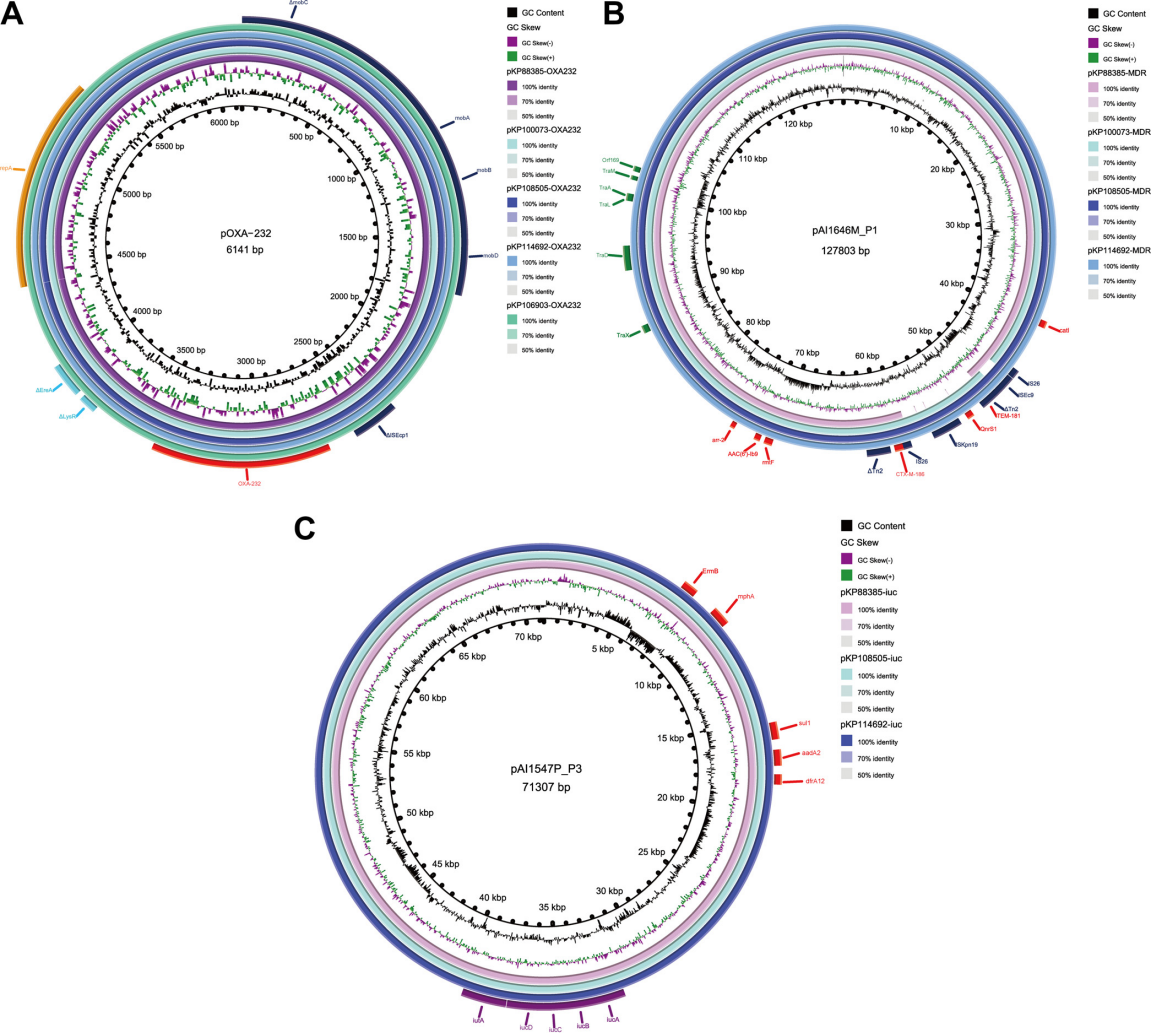
Comparison of the plasmids in four ST231 Klebsiella pneumoniae isolates from this study. (A) 6.1 kb ColKP3-type plasmid pOXA-232 bearing *bla*_OXA-232_. (B) 127.8 kb IncFIB/FII(K)-type plasmid p-MDR bearing multiple resistance genes and conjugative transfer genes. (C) 71.3 kb IncFIA/FII-type plasmid (p-iuc) harboring *iuc5* and multiple resistance genes. Antimicrobial resistance genes, virulence genes, mobile genetic elements, and conjugative transfer genes are shown as red, purple, dark blue, and green, respectively.

All of the ST231 isolates harbored three plasmids with approximate plasmid sizes of 127, 71, and 6 kb, with the exception that KP100073 only harbored two plasmids (approximately 127 and 6 kb). *bla*_OXA-232_ was located in a 6.1 kb ColKP3-type plasmid in all ST231 isolates, and it was 99.9% identical to the previously reported 6.1 kb *bla*_OXA-232_-bearing plasmid pOXA-232 (accession number JX423831.1) in the Escherichia coli and K. pneumoniae strains that were recovered from three patients who were transferred from India to France, with 100% coverage (Fig. 1A). This plasmid contained nine open reading frames (i.e., RepA, MobA, MobB, MobD, ΔMobC, ΔISEcp1, *bla*_OXA-232_, ΔLysR, and ΔEreA). The results of mating-out assays indicated that it was not transferable to Escherichia coli EC600 via conjugation. The 127.8 kb IncFIB/FII(K)-type plasmid (p-MDR) in three ST231 isolates (KP100073, KP108505, and KP114692) harboring multiple resistance genes, namely, *bla*_TEM-181_, *qnrS1*, *bla*_CTX-M-186_, *rmtF*, *acc*(*6′*)*-Ib9*, *arr-2*, and *catI*, as well as the conjugative transfer gene cluster *tra*, was 99.9% identical to the previously reported plasmid pAI1646M_P1 (accession number NZ_CP079643.1) that was isolated from a ST231 K. pneumoniae strain in India, with >98% coverage. Compared with these three isolates, the plasmid pKP88385-MDR in KP88385 lacked an 8.6 kb region bearing *bla*_TEM-181_-and *qnrS1*, which was surrounded by numerous mobile genetic elements (Fig. 1B). The *iuc5* was located in a 71.3 kb IncFIA/FII-type plasmid (p-iuc) in three ST231 isolates (KP88385, KP108505, and KP114692), which was absent in the isolate KP100073 and 99.9% identical to the previously reported plasmid pAI1547P_P3 (accession number NZ_CP079609.1) from a ST231 K. pneumoniae strain in India, with 100% coverage. This plasmid harbored multiple resistance genes (*ermB*, *mphA*, *sul1*, *aadA2*, and *dfrA12*) and a virulence gene (*iuc*5), representing genotypic antimicrobial resistance (AMR)-virulence convergence (Fig. 1C).

**FIG 2:**
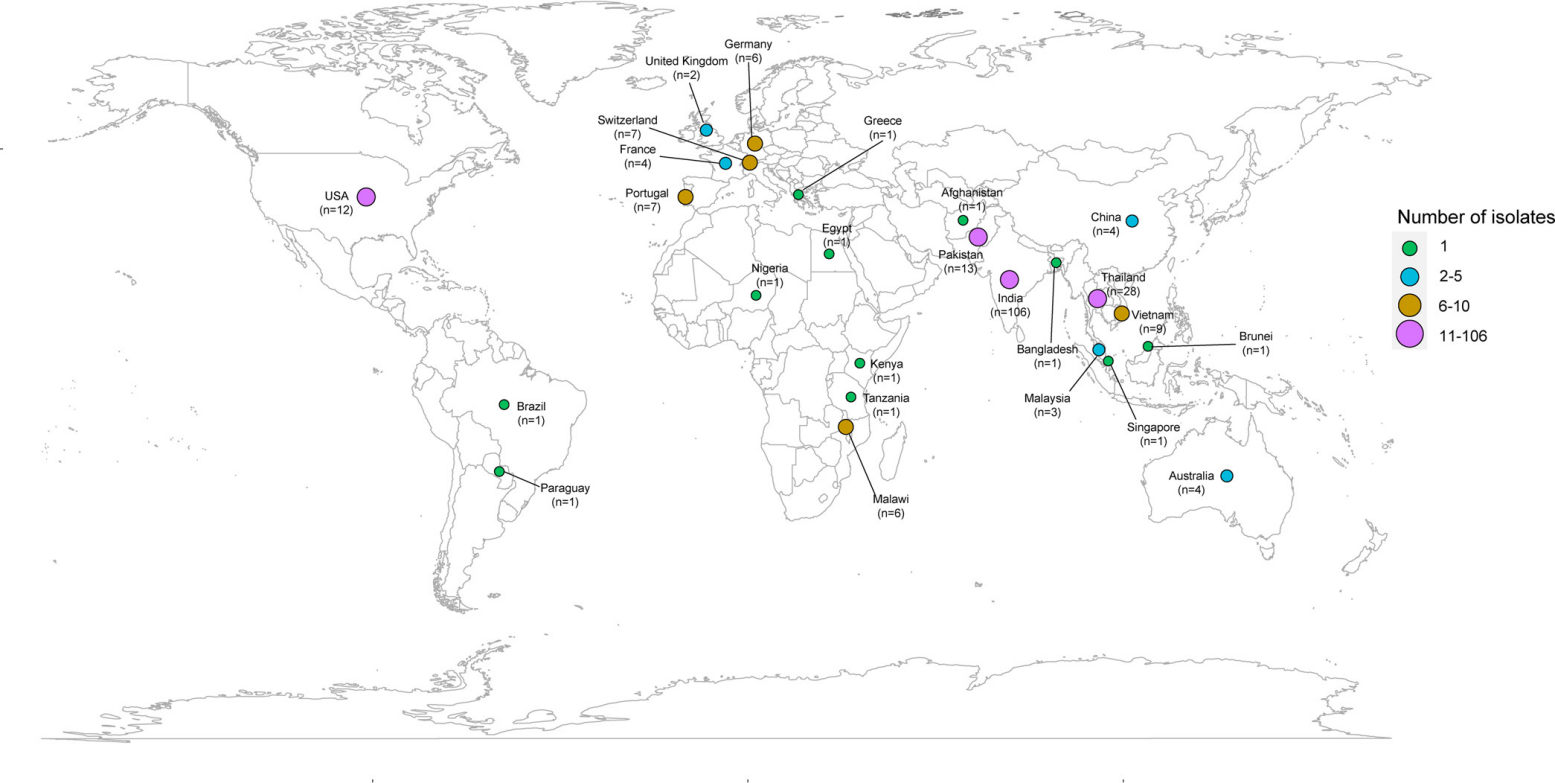
Geographical distribution of 231 ST231 K. pneumoniae isolates. The circles that represent countries with ST231 isolates are marked on the map by background color fill. The geographical origins of 19 ST231 isolates were unknown, and these are not shown in the image.

**FIG 3:**
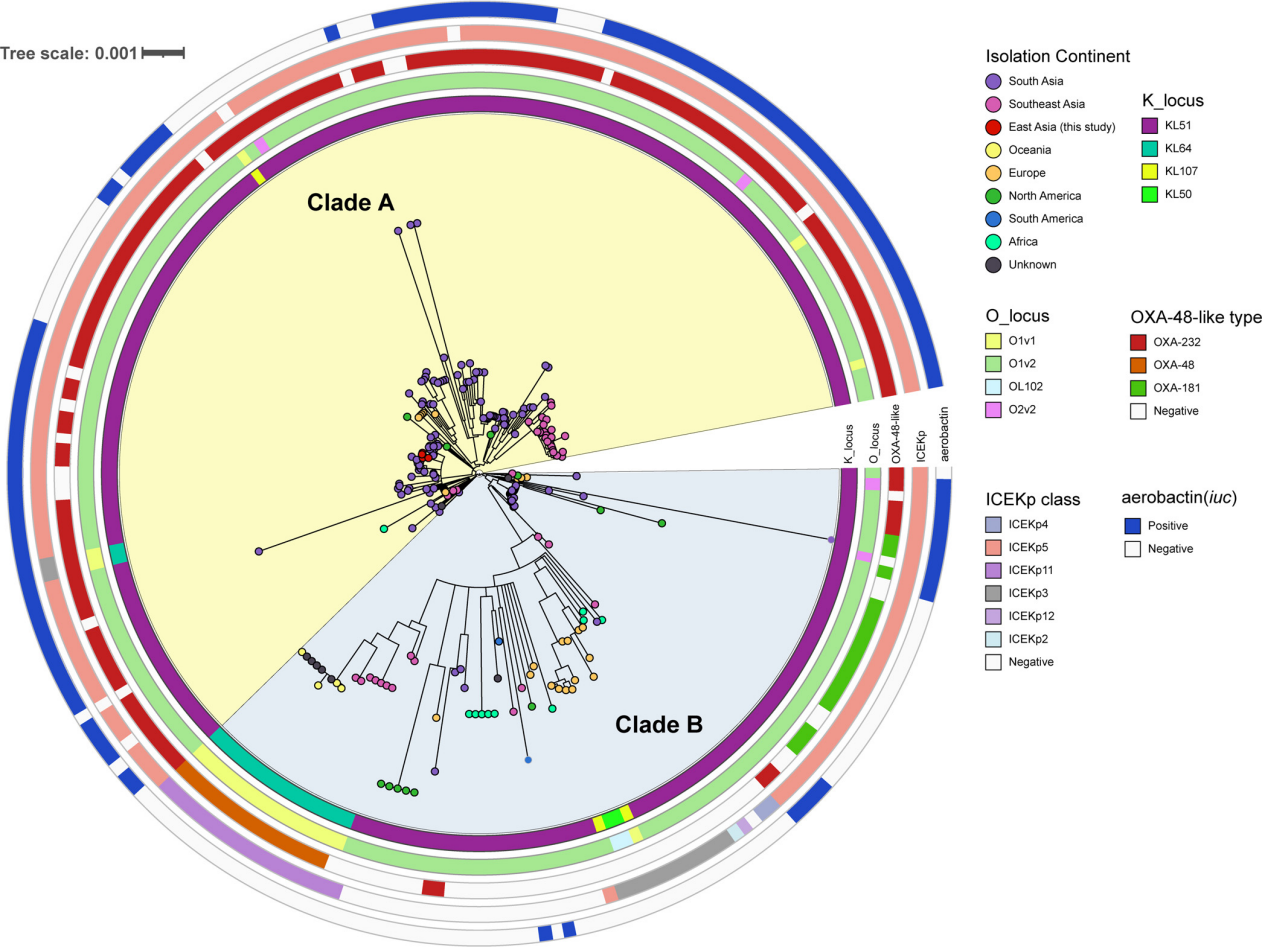
Phylogenetic structure of global 231 ST231 strains. The capsular locus, lipopolysaccharide biosynthesis locus, carbapenemase gene *bla*_OXA-48-like_ type, mobile genetic element ICEKp type, and the presence of the aerobactin *iuc* gene cluster were mapped on the tree (from the inner circle to the outer circle).

To characterize the genetic features of these four ST231 K. pneumoniae strains, we comparatively analyzed publicly available ST231 genomes (*n* = 227) from 24 regions. The global ST231 clones were obtained from India, Thailand, Pakistan, the USA, and other countries, with widespread geographic distributions (Fig. 2). A phylogenetic tree was constructed based on the core SNPs. The phylogenetic tree revealed that the 231 ST231 genomes were divided into two main clades (Fig. 3). The larger clade, namely, clade A, contained 141 genomes, mostly originating from south Asia, southeast Asia, and other regions, such as east Asia and North America. In this clade, the majority of ST231 isolates harbored the capsular locus KL51, lipopolysaccharide biosynthesis locus O1v2, carbapenemase gene *bla*_OXA-232_, mobile genetic element ICEKp5, and aerobactin *iuc* gene cluster. Notably, four ST231 isolates from this study were found in clade A and gathered with strains from south Asia (especially India). Clade B contained 90 genomes and originated from multiple regions. Furthermore, in a subclade of clade B, the ST231 isolates harbored specific KL64, O1v1, *bla*_OXA-48_, and ICEKp11, whereas *iuc* was absent.

**FIG 4:**
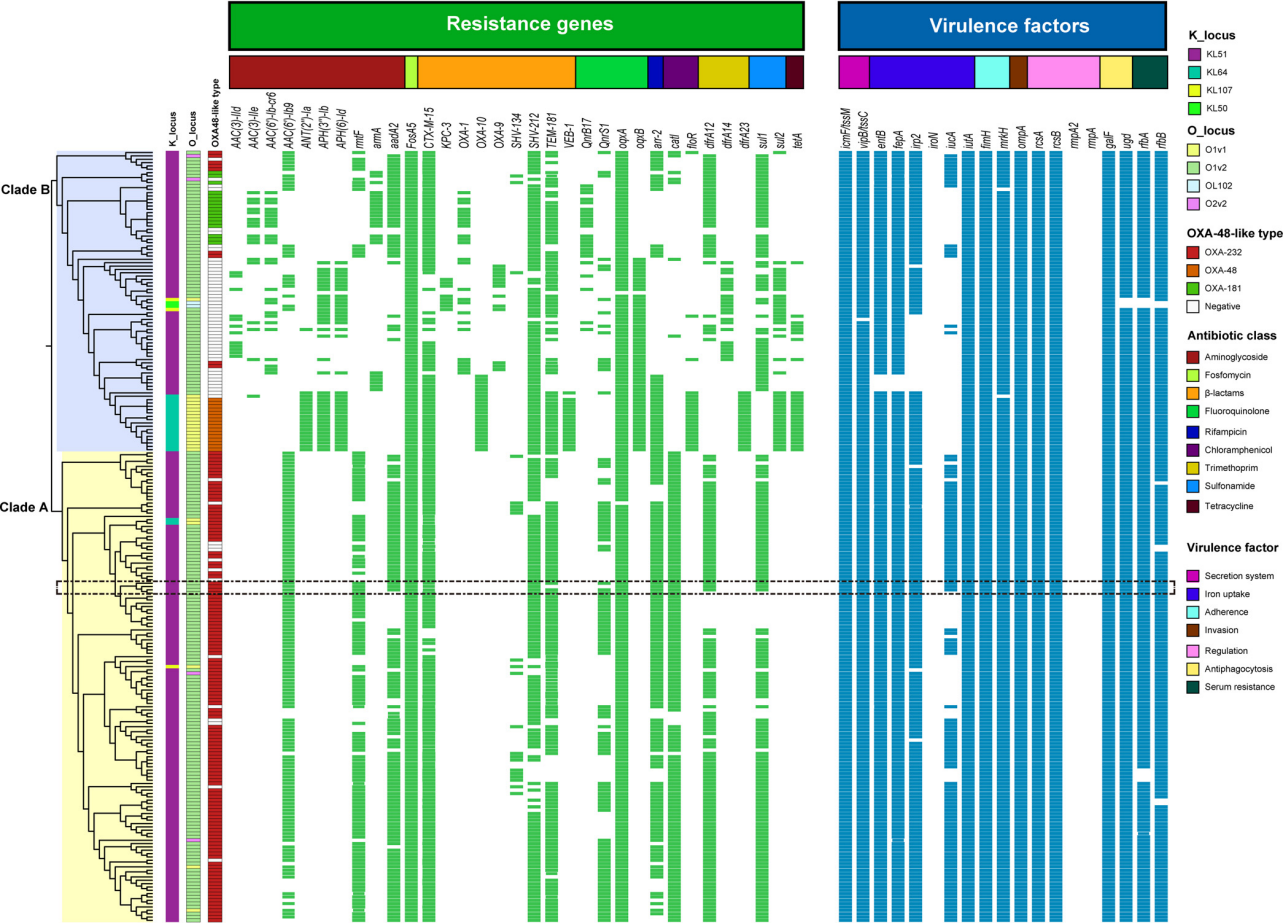
Distributions of antimicrobial resistance genes and virulence factors in the global 231 ST231 strains. The isolates from this study are indicated by the dotted box.

To compare these four ST231 isolates with those from other regions, we investigated the presence of virulence genes and resistance genes. The gene profiles of the isolates in clade A were much more consistent than were those in clade B. The resistance genes *aac*(*3*)*-IId*, *aac* (*3*)*-IIe*, *aac*(*6′*)*-Ib-cr6*, *ant*(*2″*)*-Ia*, *aph*(*3″*)*-Ib*, *aph*(*6*)*-Id*, *armA*, *bla*_KPC-3_, *bla*_OXA-1_, *bla*_OXA-10_, *bla*_OXA-9_, *qnrB17*, *oqxB*, *floR*, *dfrA14*, *dfrA23*, *sul2*, and *tetA* were exclusively detected in clade B, whereas the virulence gene *iucA* was detected more frequently in clade A (Fig. 4). Of note, the resistance profiles of the isolates in the aforementioned subclade of clade B were highly consistent and were distinctly different from the other isolates in clade B. Moreover, neither of the two capsular polysaccharide (CPS) regulator genes (*rmpA* and *rmpA2*) nor the siderophore salmochelin *iroN* gene was detected in all of the ST231 clones.

**FIG 5:**
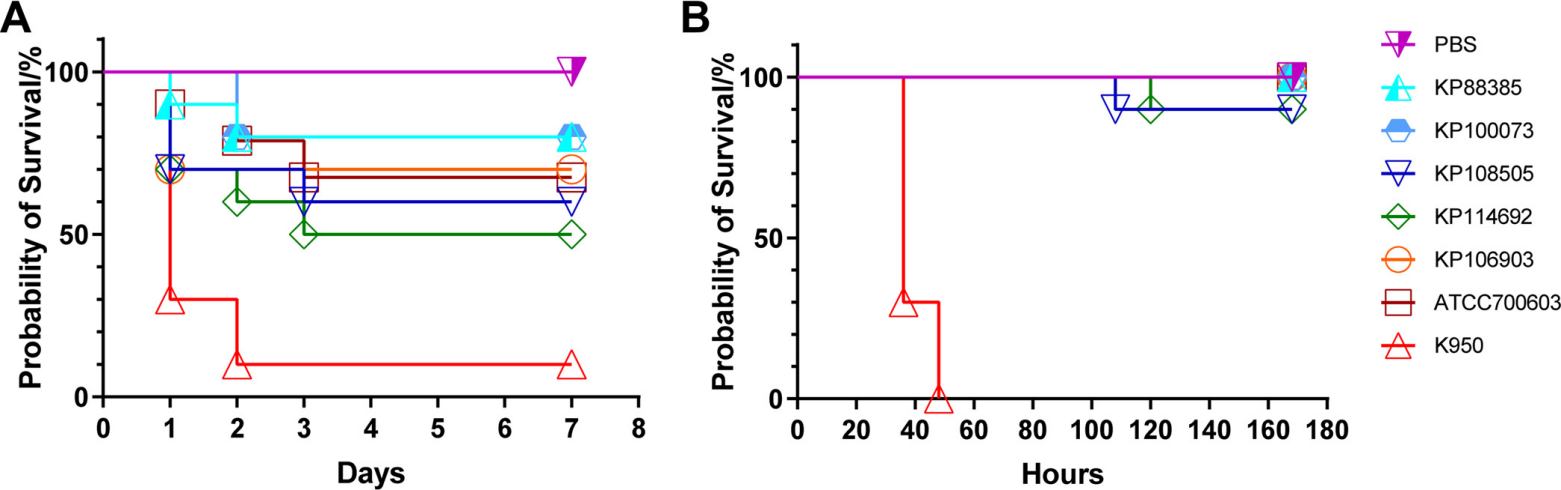
Virulence potential of OXA-232-producing ST231 and ST15 isolates in the study. (A) Survival curves in the G. mellonella infection model. 10 larvae of each group were injected with 20 μL 10^6^ CFU/mL of the strains. The survival rate over 7 days was measured. (B) Murine survival curves in the intraperitoneal infection model. 10 mice from each group were injected intraperitoneally with 10^5^ CFU of the strains. The murine survival rate over 7 days was measured.

To determine the virulence potential of the ST231 K. pneumoniae strains in the study, G. mellonella infection and mouse intraperitoneal infection models were constructed. The strain K950, belonging to K1 serotype, which was famous for its hypervirulence, as well as the classic strain ATCC700603 were used for comparison. In a G. mellonella infection model (Fig. 5A), the strain K950 displayed much higher mortality than did the ST231 and ST15 strains (*P* < 0.05), whereas no significant difference in the mortality of the larvae was observed between the classic ATCC700603 and the strains of ST231 and ST15 (*P* > 0.05). In a mouse infection model, as shown in Fig. 5B, mice infected with strain K950 all died within 48 h (*n* = 10), whereas those infected with the ST231 and ST15 strains were still alive until 108 h (*P* < 0.05). All of the results of the virulence assays indicated that the ST231 and ST15 strains in the study were not highly virulent, despite the presence of the aerobactin *iuc* gene cluster.

## DISCUSSION

Klebsiella pneumoniae, as a vital human pathogen, represents increasing multidrug-resistance, particularly to the third-generation cephalosporins and carbapenems (16). OXA-232 carbapenemase, being a critical resistance mechanism against carbapenems, has recently been increasingly reported. Notably, in China, the identified OXA-232-producing K. pneumoniae isolates almost belonged to ST15 and were not hypervirulent, despite harboring a virulence plasmid (11, 17). In this study, we first reported on a hospital outbreak of the OXA-232-producing MDR K. pneumoniae of ST231 in Yunnan, China, and we investigated the genomic features and clonal origins of these ST231 K. pneumoniae isolates.

ST231, an epidemic MDR K. pneumoniae clone in south and southeast Asia (18, 19), was increasingly found in other regions, such as Europe (20). The core genomes of the four ST231 isolates in this study differed by only a few SNPs (*n* = 9 to 16), indicating that the ST231 strains were closely related. All of these isolates displayed a multidrug resistance phenotype, which meant that there were few effective antibiotics available. The *bla*_OXA-232_ gene of the ST231 isolates was located in a 6.1 kb ColKP3-type plasmid, which was found in almost all of the OXA-232-producing K. pneumoniae isolates, belonging to various STs, including ST15, the most common OXA-232-producing clone in China (21, 22). Furthermore, a MDR plasmid with conjugative transfer genes was found in ST231 isolates, demonstrating the potential threat of the horizontal transfer of resistance genes. Of note, in the pKP88385-MDR, an 8.6 kb region bearing *bla*_TEM-181_-and *qnrS1* was lost, which may be caused by surrounding mobile genetic elements. Moreover, the presence of an AMR-virulence convergence plasmid harboring multiple resistance genes and virulence genes encoding aerobactin, which served as an important biomarker for the identification of hypervirulent isolates (23), suggested that the ST231 isolates might display a virulent phenotype in addition to the MDR phenotype.

To investigate the genomic features and the clonal origin of these ST231 K. pneumoniae isolates, we comparatively analyzed publicly available ST231 genomes from 24 regions. We separated the global ST231 K. pneumoniae isolates into two clades, and the ST231 isolates from this study were found in clade A and gathered with strains from south Asia (especially India). The ST231 isolates in clade A almost harbored identical KL51, O1v2, and ICEKp5, and a great difference of gene profiles was detected between clade A and clade B. These results indicated that the Chinese ST231 clone was more closely related to the south Asia isolates and might have spread from south Asia. As Yunnan province, which is located on the border of China, is geographically adjacent to the countries of south Asia and southeast Asia, the proximity further strengthens our speculation of transnational dissemination events of ST231 clones. However, none of the patients reported recent travel abroad, and we failed to detect any obvious route of transmission for these ST231 isolates. Further research and surveillance are needed to better understand the potential effect and transmission of this new type of K. pneumoniae in China.

It is known that ST15 isolates were not found to be hypervirulent, despite the presence of a virulence plasmid carrying *rmpA2* and the *iuc* gene cluster (11). We sought to determine the *in vivo* virulence potential of the ST231 strains due to the presence of *iuc* gene cluster, which was considered to be a promising marker of the hypervirulent phenotype, compared with *rmpA2* (24). Like ST15, the ST231 strains in the study were found to harbor significantly weaker virulence potential in G. mellonella and mouse intraperitoneal infection models than were the K1 hypervirulent isolate K950, thereby indicating that the OXA-232-producing clones of ST231 and ST15 in the study were hypovirulent and that the presence of the *iuc* gene cluster alone should not be used to predict the hypervirulence phenotype.

In conclusion, we report here the first occurrence in China of a MDR K. pneumoniae ST231 clone, which may have spread from south Asia, thereby representing a critical and worrying step toward the rise of another epidemic clone as a threat to public health. Active surveillance for OXA-232-producing K. pneumoniae should be enforced to restrict the transmission of these MDR clinical isolates, especially for immunocompromised patients.

## MATERIALS AND METHODS

### Ethics statement.

All of the animal assays were approved by the Institutional Animal Care and Ethics Committee at The First Affiliated Hospital of Zhejiang University, School of Medicine (reference number: 2022-835).

### Bacterial isolates, clinical features, and antimicrobial susceptibility testing.

Five nonduplicate carbapenem-resistant K. pneumoniae (CRKP) clinical isolates (KP88385, KP100073, KP106903, KP108505, and KP114692) were collected by the Blood Bacterial Resistant Investigation Collaborative System (BRICS) program, incorporating a total of 52 hospitals (23 tertiary hospitals and 29 non-tertiary hospitals) and covering 18 provinces in mainland China, with only strains isolated from blood samples being collected for study in the BRICS program (25). The OXA-232 β-lactamase gene of these isolates was verified via specific PCR and sequencing (pOXA232F: CGGTAGCAAAGGAATGGCAA; pOXA232R: TCGAGCATCAGCATTTTGTC). KP106903 was recovered from the First Affiliated Hospital of Zhejiang University, School of Medicine, and the other four were from the Second People's Hospital of Yunnan Province. The clinical characteristics of the patients who were infected with OXA-232-producing K. pneumoniae were retrospectively analyzed. The agar dilution method was used to determine the MICs of ceftazidime, cefuroxime, cefepime, imipenem, meropenem, ciprofloxacin, ampicillin, amikacin, gentamicin, cefoperazone-sulbactam, sulfamethoxazole-trimethoprim, and chloramphenicol, and the broth microdilution method was used for colistin and tigecycline, according to the standard protocols of the Clinical and Laboratory Standards Institute (CLSI) guidelines (26). The results were interpreted based on the breakpoints of the CLSI, except for tigecycline, which followed the criteria of the United States Food and Drug Administration (FDA) (https://www.fda.gov/drugs/development-resources/tigecycline-injection-products). All of the investigation protocols in this study were approved by the Institutional Animal Care and Ethics Committee at The First Affiliated Hospital of Zhejiang University, School of Medicine (reference number 2022-835). Informed consent was waived because this study was retrospective and observational in nature, primarily investigated bacteria, and required no interventions to be conducted with patients.

### String test.

All isolates were inoculated onto agar plates including 5% sheep blood and were incubated at 37°C overnight. The string test was performed by stretching the bacterial colony on agar plates using a bacteriological loop. The string test was deemed positive when a colony stretched at least 5 mm.

### Whole-genome sequencing and multilocus sequence typing (MLST).

We sequenced all five isolates using an Illumina Hiseq2500 instrument (Illumina, https://www.illumina.com) with 2 × 125-bp paired-end libraries. We performed the *de novo* assembly of the short-read data by using SPAdes 3.14.1 (27). The sequence type of each isolate was acquired via MLST (https://cge.food.dtu.dk/services/MLST/). MinION sequencing (Oxford Nanopore Technologies Inc., UK) was further performed on the four isolates of ST231. Complete genomes of the four ST231 isolates were generated using hybrid assembly with Illumina and Nanopore reads using the Unicycler v0.4.0 tool (28), and they were annotated using the RAST server (https://rast.nmpdr.org). A pairwise SNP analysis of the four ST231 strains, based on their complete genomes, was conducted using CSI Phylogeny 1.4 (https://cge.food.dtu.dk/services/CSIPhylogeny/). The K-type and O-type were identified by using Kleborate (29). Insertion sequences were identified using ISsaga (http://issaga.biotoul.fr/issaga_index.php) and the IS Finder database (https://www-is.biotoul.fr/). We deposited the five genome sequences in GenBank under PRJNA838446 (Table S1).

### Phylogenetic analysis.

Publicly available ST231 K. pneumoniae genomes (*n* = 227) were obtained from PATRIC (https://www.patricbrc.org/). The details of these isolates are listed in Table S1. Snippy v4.6.0 (https://github.com/tseemann/snippy) was used to identify the SNPs for the ST231 core genome. The complete genome of the ST231 isolate MSB1_8A-sc-2280397 (GenBank assembly accession: GCA_003432165.1) served as a reference. The recombined regions within the core genome were detected using Gubbins v2.4.1 (30). The phylogenetic tree for the ST231 isolates was constructed using the core SNPs from the recombination-free core-genome alignment with Fasttree 2.1.11, using the default settings (http://www.microbesonline.org/fasttree/).

### Comparison of antimicrobial resistance genes and virulence factors among ST231 isolates.

The antimicrobial resistance genes and the virulence factors were identified by using ABRicate v1.0.0 (https://github.com/tseemann/abricate), based on the CARD (31) and VFDB (32) databases with the default settings.

### Transferability of the *bla*_OXA-232_ gene.

The transfer of the *bla*_OXA-232_ gene was attempted via mating-out assays, using rifampicin-resistant Escherichia coli EC600 as a recipient strain. Imipenem (0.12 mg/L) and rifampicin (700 mg/L) were used for the selection of the transconjugants.

### Virulence assays.

All of the animal experiments were approved by the Institutional Animal Care and Ethics Committee of the First Affiliated Hospital of Zhejiang University, School of Medicine (reference number: 2022-835). Galleria mellonella (G. mellonella) and mouse intraperitoneal infection models were established as previously described (33, 34). Briefly, for the former, dilutions of bacteria (10^6^ CFU/mL) were prepared in 1× phosphate-buffered saline (PBS). 10 larvae, weighing between 200 and 250 mg, were randomly selected for each isolate. Subsequently, 20 μL of bacteria were injected into the second left proleg. Larvae that had been injected with 20 μL of PBS were used as a control. The insects were incubated at 37°C in the dark and were observed at 24 h intervals for 7 days. For the latter, 5-week-old BALB/c-nude female mice were used for this study. The mice were injected intraperitoneally with 1 × 10^5^ CFU of isolates, which were resuspended in 100 μL of 1× PBS (10 mice from each group were injected). After the inoculation, the physical condition of each mouse was monitored and recorded every 12 h. The hypervirulent K. pneumoniae strain K950 (ST23, K1 serotype, hypermucoviscosity) (35) and the classic K. pneumoniae strain ATCC700603 were used as controls of high and low virulence strains, respectively.

### Statistical analysis.

All of the experiments were conducted independently, with at least three replicates, on different days. The mortality of the G. mellonella and the mice was assessed via a Kaplan-Meier analysis and a log-rank test, using GraphPad Prism 9.

### Data availability.

The whole-genome sequences of all of the collected K. pneumoniae isolates have been deposited in the GenBank database under the BioProject accession number PRJNA838446.
